# A Glance Into Healthcare Delivery During COVID-19 Pandemic: A Survey Among Turkish Medical Doctors

**DOI:** 10.3389/fmed.2022.890417

**Published:** 2022-07-19

**Authors:** Irem Karaman, Selin Ildir, Sevket Ozkaya

**Affiliations:** ^1^School of Medicine, Bahcesehir University, Istanbul, Turkey; ^2^Department of Pulmonary Medicine, Faculty of Medicine, Bahcesehir University, Istanbul, Turkey; ^3^Department of Pulmonary Medicine, Faculty of Medicine, Altinbas University, Istanbul, Turkey

**Keywords:** COVID-19, healthcare, non-COVID-19 diseases, delayed diagnosis, telemedicine

## Abstract

The coronavirus 2019 (COVID-19) pandemic had an enormous impact on healthcare delivery globally. We conducted a cross-sectional online survey in Turkey to evaluate the impact of COVID-19 on healthcare services in Turkey. A 35-item anonymized online survey was completed by HCPs (medical doctors, MD) who continued their clinical practice during the COVID-19 pandemic in Turkey, regardless of their specialties or degrees. Overall, 209 HCPs participated in the study. Forty-two percent of the participants stated that their current workload intensity has been increased compared with the pre-pandemic era. More than half of the participants (54.6%) were using telemedicine services during their clinical practice, however, the effectiveness of telemedicine for first-time patients and follow-up patients was rated as low. The majority of participants (59.3%) reported that during the peak period of the pandemic, they encountered only a small variety of cases, other than COVID-19. Fifty-two percent of the participants agreed that they occasionally had patients who received misdiagnosis in the first admission due to the suspicion of a possible COVID-19 infection predominating the diagnostic process (eg., not excluding COVID-19 even though the PCR test is negative). For the distribution of possible late-diagnosed diseases, 25.8% of HCPs selected chest diseases, followed by infectious diseases, heart diseases, and cancer. In general, participants agreed that there was an increase in the negligence in the follow-up of various diseases and/or complication rates due to COVID-19 pandemic. Sixty percent of the HCPs agreed that HCPs are being much more rigorous to diagnose/treating COVID-19 than other important diseases. Fifty-seven percent of the participants stated that the diagnosis and follow-up of chronic diseases are affected, while 57.9% of the HCPs stated that some diseases that show similar signs and symptoms as COVID-19 are not diagnosed correctly during COVID-19 pandemic. Findings from this study emphasize that COVID-19 pandemic has significantly caused delayed diagnoses and interruption in the management of chronic diseases, and also increased the risk of missing out the diagnosis of non-COVID-19 diseases. The study genuinely aims to yield the floor to a permanent improvement in post-pandemic clinical management and it also shows the need for a focused approach in distinct areas of medical care. Policymaking is required to drive changes to better support HCPs in Turkey.

## Introduction

The emergence of SARS-CoV-2 capsized the global health services extensively, leading to a COVID-19-centered approach for the health facilities as of March 11, 2020, after the declaration of the pandemic by the World Health Organization (WHO) (1). On the same day, the first case of SARS-CoV-2 infection in Turkey was confirmed by the Turkish Ministry of Health (2). To date, COVID-19 keeps its place as one of the primary focuses of the Turkish health system with 15,061,376 official cases by May 22, 2022 (2).

COVID-19 is a multisystemic disease with a wide spectrum of clinical and radiological presentations that vary according to the medical history and the immunocompetency of the patient (3). The divergence in respiratory, cardiovascular, and immunological phenotypes of COVID-19 may either present as mild-to-moderate disease or end-stage organ damage. It may produce non-specific, multi-systemic findings such as fever, cough, fatigue, dyspnea, diarrhea, headache, rhinitis, or skin rash (4). When the post-infectious syndromes that stem from pulmonary fibrosis or cardiovascular stress are added to the list, the situation alarms clinicians who practice during the **pandemic** era to utilize a detailed elaboration to identify patient needs and meticulously concentrate on their different manifestations of COVID-19 (5). Furthermore, both the government-related and self-implemented restrictions in the community hinder the visits to health-service centers, leading to an **inaccurate** diagnosis of many diseases, interrupted treatment and control schedules for the frequently seen pre-pandemic, non-communicable diseases and infections other than COVID-19. Chudasama et al. point out that some crucial morbidities and various disease spectrums such as diabetes, hypertension, **chronic obstructive pulmonary disease (COPD)**, and heart failure are often overlooked due to diminished access to healthcare (6). Fekadu et al. emphasize that since the physical examination opportunities and in-person contact with the healthcare personnel have been lost for many diseases as a result of nationwide restrictions, the routine analysis of some chronic diseases and the individual care for community-acquired diseases have been impaired, leading to a targeted yet concerning approach against COVID-19 (7). The impact of the COVID-19 pandemic on health services can be categorized into three main aspects: 1. delayed diagnoses of non-COVID-19 diseases due to the interference of screening and follow-up, 2. neglected acute/subacute/chronic diseases that course without identification, and 3. misdiagnoses due to mimicry of certain signs and symptoms and a COVID-19 centered clinical approach (8, 9). The negative effects of the catastrophic COVID-19 era for HCPs can be listed as increased infection risk, social detachment from beloved ones and the rest of the society, and economical stress, all leading to professional burnout (10). Alarmingly, the burnout in healthcare systems brings more about medical mismanagement and inaccuracy and decreases the quality of care that the community receives.

Turkey may be mentioned as one of the countries that were highly affected by COVID-19 despite its abundance in medical systems. Two peak periods are defined as being first in April 2020 and eventually in April 2021 (10, 11). Throughout the country, the term “normalization period” is used for the diminishing in case numbers that enable somewhat loosening in government restrictions and lockdowns. Restrictions were removed for all age groups in July 2021, pointing to the beginning of a fully “normalized” time interval. The vaccination periods can be summarized as being initially Coronavac(Sinovac) for HCPs and the elderly in January 2021, followed by the accelerated application rates on May 20, 2021, via the addition of Pfizer/BioNTech vaccine. Since then, every day, exceeding one million citizens were vaccinated. According to Our World in Data, by the end of August 2021, 57 percent of the population was at least partially vaccinated, and 43 percent of the population was fully vaccinated, and by May 2021, 68 percent of the population was at least partially vaccinated, and 62 percent of the population was fully vaccinated (11, 12).

This study aimed to investigate the ongoing impact of COVID-19 on healthcare services in Turkey in terms of the diagnostic workup, delayed diagnoses, misdiagnoses, and factors affecting the quality of care for diagnosing, following and treating diseases, as well as reporting the healthcare professionals'(HCP) perspective. Additional data about the impact of COVID-19 on the mental health of HCP, fear of COVID-19, qualification of healthcare services, and potential reasons behind interrupted healthcare services during the COVID-19 pandemic were also reported. The secondary aims of the study were to determine which diseases had difficulties diagnosing during the pandemic, and to measure the impact of the pandemic on the adequacy of hospital services. It was also among the objectives to understand whether the failures in the diagnosis and treatment in the pandemic era occur within the framework of the negligence of the patient or the healthcare provider, regarding the diseases with signs and symptoms that can be confused with COVID-19 or other various non-communicable diseases that require control-examination.

## Materials and Methods

### Design, Study Population, and Online Questionnaire

We conducted a national cross-sectional online survey in Turkey between January—February 2022. A 35-item anonymized online survey targeting the HCPs (medical doctors, MD) who continued their clinical practice during the COVID-19 pandemic in Turkey was designed by the authors, specifically for this study, based on the emerging literature and consultation with local experts. The participants were employed in a range of settings including general practice, community health services, operating rooms, occupational health, and university hospitals. The majority of the participants were the ones who were seeing COVID-19 patients in either outpatient or inpatient clinics(e.g, COVID-19 clinic/service). HCPs who were not currently seeing patients, and HCPs other than MDs(e.g, pharmacists who mistakenly filled the form) were excluded from the study **(*n*** **=** **3)**. Prior to dissemination, the survey was tested by a group of HCPs for the time to complete and to ensure no questions were distressing. Some minor formatting and language changes were made to enhance survey readability and flow. The survey was administered in Turkish for better understanding. In addition to 35-item multiple-choice questions, there was an open-ended question for HCPs who would like to state their opinion at the end of the survey. The aim of an open-ended question for additional thoughts was to eradicate the possibility of a leaded or overseen point of view of the survey. The open-ended question did not have any context or a possible source of bias.

The electronic link of the survey was posted on social media (including Twitter, and LinkedIn), websites (social platforms that were used by HCPs), and mailing lists belonging to HCP networks. The posts were sharable to facilitate snowball sampling. All participants consented to participate in the study prior to survey completion. This study was approved by the Republic of Turkey Ministry of Health and Ondokuz Mayis University Institutional Review Board (No: 2021/599) with respect to its scientific content.

The questionnaire was programmed using the open-sourced web survey application, Google Forms, and consisted of four sections: (a) Demographic characteristics; (b) Impact of the COVID-19 pandemic on clinical practice; (c) Impact of COVID-19 on patient diagnosis and follow-up processes; (d) Feelings and fears regarding COVID-19 pandemic. Each section consisted of either multiple-choice questions or Likert-scale questions on a 5-point or 10-point scale from certainly disagree (1) to strongly agree(5/10). The Likert-scale questions were constituted by analyzing the similar survey studies in the literature to decide counterparts. Data from other sections of the survey addressed discrete and different research questions and so is reported elsewhere. Open-ended data from a different part of the questionnaire was also reported.

### Statistical Analysis

The data of this study was transferred to IBM SPSS 25 (IBM Statistical Package for Social Sciences) and assessed. The descriptive statistics of categorical variables were presented both numerically and in percentages. The assumptions of normality were checked for all variables by inspecting the distribution visually using histograms and probability graphics, and through analytical methods (Kolmogorov-Smirnov/Shapiro-Wilk tests). In the comparison process, since the descriptive analysis did not have a normal distribution, data were given as median (min-max). The descriptive analysis of numerical variables was presented as both median (min-max) / mean ± Standard Deviation. Analysis of categorical variables was done based on the comparison via Continuity Correction and Pearson Chi-Square test, supported by cross tables. The degree of relation between categorical values was determined by the contingency coefficient. For the decision of significant subgroups, Benferonni correction was applied. In two-group numerical variables that were non-normally distributed, the Mann-Whitney U test was used as the statistical method; whereas Kruskal Wallis-H was preferred for more than two group numerical variables, followed by a pairwise comparison for the significant ones. A *p*-value < 0.05 was accepted as statistically significant.

## Results

### Participant Characteristics

Overall, 209 HCPs participated in the study. Fifty-four percent of the participants were female, and the mean age was 41. The majority of participants were attending doctors, followed by residents, associate professors, assistant professors, professors, and general practitioners. About a quarter of the participants were lung diseases specialists, 33% of the participants were from other internal departments and 16.7% of participants were from surgical departments. On average, participants had 15 years of professional experience. Sixty-eight percent of the participants worked in the COVID-19 service during the pandemic, and 40.7% of the participants had COVID-19. The additional participant demographics were provided in Table 1.

**Table 1:** Distribution of demographical and COVID-19 related characteristics.

**Variables**		**Descriptive statistics *N* (%)**
Age (median,min-max) (mean ± *SD*)		41 (24–79) / 41.68 ± 10.55
Gender	Female	114 (54.5%)
	Male	95 (45.5%)
Academic title	Attending doctor	59 (28.2%)
	Resident doctor	42 (20.1%)
	Assistant professor	20 (9.6%)
	Associate professor	20 (9.6%)
	Professor	13 (6.2%)
	General practitioner	11 (5.3%)
	Research associate	7 (3.3%)
	No response	37 (17.7%)
Department	Chest Diseases	51 (24.4%)
	Other Medical Sciences^**^	47 (22.5%)
	Surgery^***^	35 (16.7%)
	Internal Medicine	26 (12.4%)
	Emergency	6 (2.9%)
	Infectious Diseases	4 (1.9%)
	Intensive Care	3 (1.4%)
	No response	37 (17.7%)
Professional experience (years) (median,min-max) (mean ± *SD*)		15 (1–55)/ 16.41 ± 10.65
Average number of patients that you encounter per day(median,min-max) (mean ± *SD*)		40 (2–1200)/ 51.64 ± 117.6
Have you worked in the COVID-19 service during the pandemic?	Yes	143 (68.4%)
	No	66 (31.6%)
Have you had COVID-19?	Yes	85 (40.7%)
	No	124 (59.3%)
Medical unit	Family health center	14 (6.7%)
	Public hospital	111 (53.1%)
	Private hospital	55 (26.3%)
	Training&Research hospitals/University hospitals	29 (13.9%)
The main area of work	Outpatient clinic	143 (68.4%)
	Intensive care unit	24 (11.5%)
	Inpatient clinic/Service	30 (14.4%)
	Operating room	12 (5.7%)

** *Family Medicine, General Practitioner, Occupational Medicine, Paediatrics, Radiology, Psychiatry, Neurology, Clinical Biochemistry, Dermatology, Physical Therapy and Rehabilitation, Oncology*.

*** *General Surgery, Otorhinolarngology, Obsteterics and Gynecology, Anesthesiology and Reanimation, Thoracic Surgery*.

### Impact of the COVID-19 Pandemic on Clinical Practice: Disease Diagnosis and Follow-Up

The data regarding the impact of the COVID-19 pandemic, telemedicine services, and COVID-19 vaccination on clinical practice during the COVID-19 pandemic was presented in Table 2. Forty-two percent of the participants stated that their current workload intensity has been increased compared with the pre-pandemic era. More than half of the participants were using telemedicine services during their clinical practice, however, the perceived effectiveness of telemedicine for first-time patients and follow-up patients was rated as 2(1: Not effective, 10: Highly effective). Participants gave 6 out of 10 on average for the time they spare for their patients and various disease spectrums who applied after the restrictions were lifted (mean Likert score: 6). Forty-two percent of the participants expressed that they highly prioritize suspecting and diagnosing COVID-19 during their clinical practice. The majority of participants (59.3%) reported that during the peak period of the pandemic, they encountered only a small variety of cases, other than COVID-19. HCPs (70.8%) reported that the normalization period facilitated increased rates of physician applications, ensured patient diagnosis and follow-up, and increase non-COVID-19 case diversity, which was further accelerated with the COVID-19 vaccination process.

**Table 2:** Impact of the COVID-19 pandemic, telemedicine services and COVID-19 vaccination on clinical practice during the pandemic.

**Variables**		**Descriptive Statistics *N* (%)**
Please compare the current workload intensity in the outpatient clinic/emergency room/medical unit you are assigned with with the pre-COVID-19 pandemic era (before March 2020)	Similar or equal	79 (37.8%)
	More intense before the pandemic	42 (20.1%)
	More intense after the pandemic	88 (42.1%)
Does your institution/medical unit use telemedicine services in routine clinical procedures?	Sometimes	66 (31.6%)
	Mostly	48 (23%)
	Never	95 (45.5%)
If you responded “mostly” or “occasionally” to the previous question, please rate the effectiveness of teleclinics in first-time patients (1: Not effective, 10: Highly effective) (median,min-max) (mean ± *SD*)		2 (0–10)/3.13 ± 3.37
If you responded “mostly” or “occasionally” two questions before, please rate the effectiveness of teleclinics in follow-up patients (1: Not effective, 10: Highly effective) (median,min-max) (mean ± *SD*)		2 (0–10)/ 3.28 ± 3.44
Can you spare as much time as before the pandemic for the patients who had different kinds of diseases other than COVID-19 after the restrictions were lifted? (1: Certainly not, 10: Certainly yes) (median,min-max) (mean ± *SD*)		6 (1–10)/ 5.91 ± 2.67
On average, how important is the suspicion or diagnosis of COVID-19 (meaning PCR requirement, positivity or radiological/clinical findings) by percentage (%) in the patient profile applying to outpatient clinic that you encounter in your daily practice?	0–20%	43 (20.6%)
	21–40%	35 (16.7%)
	41–60%	43 (20.6%)
	61–80%	50 (23.9%)
	81–100%	38 (18.2%)
Evaluate your monthly non-COVID-19 case diversity by comparing the peak period of the COVID-19 pandemic with the pre-pandemic era.	During the peak period of the pandemic, I encountered a small variety of cases, other than COVID-19.	124 (%59.3%)
	During the peak period of the pandemic, I encountered a wide variety of cases, other han COVID-19.	14 (6.7%)
	During the peak of the pandemic, I have not dealt with any COVID-19 patients.	12 (5.7%)
	During the peak period of the pandemic, similar or the same variety of cases applied as the pre-pandemic period.	35 (16.7%)
	During the peak of the pandemic, I only dealt with COVID-19 patients.	24 (11.5%)
Considering the normalisation period started after vaccination, evaluate the monthly non-COVID-19 case diversity between 1 and 10 by comparing it to the pre-pandemic period (before March 2020). (1: Only COVID-19, no diversity, 10: **10** Maximum diversity, similar to pre-pandemic) (median,min-max) (mean ± *SD*)		7 (1–10)/ 7.03 ± 2.11
Do you think that vaccination facilitates the patient diagnosis and follow-up processes during the COVID-19 pandemic? (1: Not at all, 5:Definitely yes) (median,min-max) (mean ± *SD*)		5 (1–5)/ 4.18 ± 1.06
With vaccination and normalization, has there been an increase in appointments for non-COVID-19 diseases?	Yes	148 (70.8%)
	No	35 (16.7%)
	No opinion	26 (12.4%)

Table 3 consisted of questions investigating the impact of the COVID-19 pandemic on patient diagnosis and follow-up processes. Fifty-two percent of the participants agreed that they occasionally had mistakenly diagnosed patients in the first admission, due to a tendency to not being able to exclude COVID-19 possibility, where the patients either might have gotten an accurate diagnosis later on or continued with the misdiagnosis. Among the possible late diagnoses, more than a quarter selected the chest diseases, followed by infectious diseases, heart diseases, and cancer. In general, participants agreed that there was an increase in the negligence in the follow-up of various diseases and/or complication rates due to COVID-19 pandemic (mean Likert score: 7/10). Sixty percent of the HCPs agreed that HCPs are being much more rigorous to diagnose/treating COVID-19 than other important diseases. Fifty-seven percent of the participants stated that the diagnosis and follow-up of chronic diseases are affected, while 57.9% of the HCPs stated that some diseases that show similar signs and symptoms as COVID-19 are not diagnosed correctly during COVID-19 pandemic.

**Table 3:** The effect of the COVID-19 pandemic on patient diagnosis and follow-up processes.

**Variables**	**Descriptive Statistics** ***N*** **(%)**	
Have you had any cases who were mistakenly diagnosed in the first admission since the suspicion of a possible COVID-19 infection in the patient predominates the diagnostic process (eg. not excluding COVID-19 even though the PCR test is negative)?	Sometimes	93 (44.5%)
	Very often	17 (8.1%)
	Never	36 (17.2%)
	Rarely	63 (30.1%)
If you answered “always”, “very often”, “sometimes” or “rarely”, which of the following is most appropriate for the interdisciplinary distribution of these diseases?	Infectious diseases	38 (18.2%)
	Cancer	21 (10%)
	Heart diseases	22 (10.5%)
	Chest diseases	54 (25.8%)
	Local manifestations of systemic diseases	15 (7.2%)
	Metabolic diseases	8 (3.8%)
	Neurological diseases	5 (2.4%)
	Musculoskeletal diseases	1 (0.5%)
	Didn't respond	45 (21.5%)
Do you think that there is an increase negligence of the follow-up of various diseases and/or complication rates due to COVID-19 pandemic? Score between 1 and 10. (1:Absolutely no 10:Absolutely yes) (median,min-max) (mean ± *SD*)		7 (1–10)/ 6.25 ± 2.31
Do you think that health-care professionals are being much more rigorous to diagnose/treat an uncertain disease like COVID-19 than to other important diseases?	Yes	127 (60.8%)
	No	54 (25.8%)
	No opinion	28 (13.4%)
Do you think that the diagnosis of diseases whose symptoms are similar to COVID-19 may have been neglected due to the timeliness of COVID-19? (Respondents are allowed to choose more than one answer since the choices include 3 scenerios for chronic diseases and 3 for COVID-19 mimicking ones.)	I think that the diagnosis and follow-up of chronic diseases are mostly missed.	34 (16.3%)
	I think that the diagnosis and follow-up of chronic diseases are missed from time to time.	86 (41.1%)
	I do not think that the diagnosis and follow-up of chronic diseases are significantly affected.	30 (14.4%)
	I think that some diseases that show similar signs and symptoms as COVID-19 are mostly not diagnosed correctly.	48 (23%)
	I think that some diseases that show similar signs and symptoms with COVID-19 are not properly diagnosed from time to time.	73 (34.9%)
	I do not think that the diagnosis of diseases with similar signs and symptoms as COVID-19 is affected.	8 (3.8%)

The effect of the COVID-19 pandemic on clinical services and healthcare, and COVID-19-related feelings and thoughts were presented in Table 4. In general, HCPs reported an increase in the rate of laboratory and radiological tests requested for diagnosis/follow-up of patients after the normalization, compared to the pre-pandemic era. Most of the participants believe that the clinical services, number of appointments, and hospital facilities became sufficient for the diagnosis and follow-up of the patients after the normalization, compared with the pre-pandemic era. While 48.8% of the HCPs thought that patient-physician communication was not affected by the COVID-19 pandemic, 38.3% of HCPs thought that COVID-19 had a negative impact on communication whereas 12.9% thought it had a positive impact. Most of the HCPs evaluate the compliance of the patients who require chronic and/or routine screening (cancer, diabetes, hypertension, asthma, COPD, etc.) from March 2020 to the present, to apply to the hospital or to comply with the controls as low-moderate(mean Likert score:4) Among the reasons for this low-to-moderate compliance, most popular opinions were the idea that health-care centers carry a higher risk of transmitting COVID-19 (79.4%), along with the inability or hesitation to visit hospitals during the pandemic (61.7%), automatic extension of medication reports, the difficulty of finding an appointment, and patient's neglect (similar or same as before the pandemic). Among the problems encountered throughout the pandemic, the major concerns were the lack of control/follow-up (69.4%), hospitalization problems (lack of space, risk of infection, etc.,) (%67.5), increase in late diagnoses (65.1%), and delayed surgical decisions (42.1%). HCPs had increased levels of fear of being infected with SARS-CoV-2, and they were essentially afraid of infecting their family and patients with SARS-CoV-2. Most of the HCPs were satisfied with the hospital services and personal protective equipment provided by their hospital. Eighty-one percent of HCPs reported that a COVID-19-centered clinical practice, which is implemented during the pandemic, adversely affected the mental health of field physicians during their diagnostic processes.

**Table 4:** Impact of the COVID-19 pandemic on clinical services and healthcare, and COVID-19-related feelings and thoughts.

**Variables**	**Descriptive Statistics** ***N*** **(%)**	
Do you think that there is an increase in the rate of laboratory and radiological tests requested for diagnosis/follow-up of patients after normalization compared to pre-pandemic (March 2020)? (0:Certainly not, 10:Certainly yes) (median,min-max) (mean ± *SD*)		8 (1–10)/ 7.12 ± 2.31
Do you think that after the normalization, the clinical services, the number of appointments and the hospital facilities are sufficient for the diagnosis and follow-up of the patients compared with the pre-pandemic era (March 2020)? (0:Certainly not, 10:Certainly yes) (median,min-max) (mean ± *SD*)		6 (1–10)/ 5.62 ± 2.53
Evaluate the patient-physician communication by comparing it with the pre-pandemic (March 2020).	More positive communication than before the pandemic	27 (12.9%)
	More negative/problematic communication than before the pandemic	80 (38.3%)
	Similar or same communication as before the pandemic	102 (48.8%)
Evaluate the **compliance** of patients who require chronic and/or routine screening (cancer, diabetes, hypertension, asthma, COPD, etc.) from March 2020 to the present, to apply to the hospital or to comply with the controls, on a scale of 1 to 10, according to the majority. (1:No control visits 10: All controls have been done without interruption) (median,min-max) (mean ± *SD*)		4 (1–9)/ 4.7 ± 1.89
Which of the following is most likely to be the reason for your answer to the previous question? (You can mark more than one reason.)	Difficulty of finding an appointment	74 (35.4%)
	Automatic extension of medication reports	94 (45%)
	Idea that health-care centers carry a higher risk of transmitting COVID-19	166 (79.4%)
	Patient's personal neglect (similar or same as before the pandemic)	54 (25.8%)
	Inability or hesitation to visit hospitals during the pandemic	129 (61.7%)
	Financial reasons	19 (9.1%)
Did you encounter any problems in the health services of non-COVID-19 diseases throughout the pandemic? (You can mark more than one.)	Difficulty of finding medications	50 (23.9%)
	Lack of control/follow-up	145 (69.4%)
	Increase in late diagnoses	136 (65.1%)
	Increasing misdiagnoses	43 (20.6%)
	Delayed surgical decisions	88 (42.1%)
	Hospitalization problems (lack of space, risk of infection, etc.)	141 (67.5%)
	Healthcare services in areas other than COVID-19 are the same as before March 2020.	11 (5.3%)
	Health services in areas other than COVID-19 are better than before March 2020.	4 (1.9%)
Are you afraid of being infected with SARS-CoV-2? (1:Not at all, 5:Absolutely yes) (median,min-max) (mean ± *SD*)		4 (1–5)/ 3.51 ± 1.36
Do you think that the hospital services and personal protective equipment provided by your hospital are sufficient? (1:Not at all, 5:Absolutely yes)		4 (1–5)/ 3.44 ± 1.32
Are you afraid of infecting your family and patients with SARS-CoV-2? (1:Not at all, 5:Absolutely yes) (median,min-max) (mean ± *SD*)		5 (1–5)/ 4.51 ± 0.98
How do you think a COVID-19-centered clinical practice, which is implemented during the pandemic, affects field physicians mentally during their diagnostic processes?	I have no opinion	18 (8.6%)
	The mental health of physicians were not affected.	8 (3.8%)
	The mental health of physicians were affected positively.	12 (5.7%)
	The mental health of physicians were adversely affected.	171 (81.8%)

**Figure 1:**
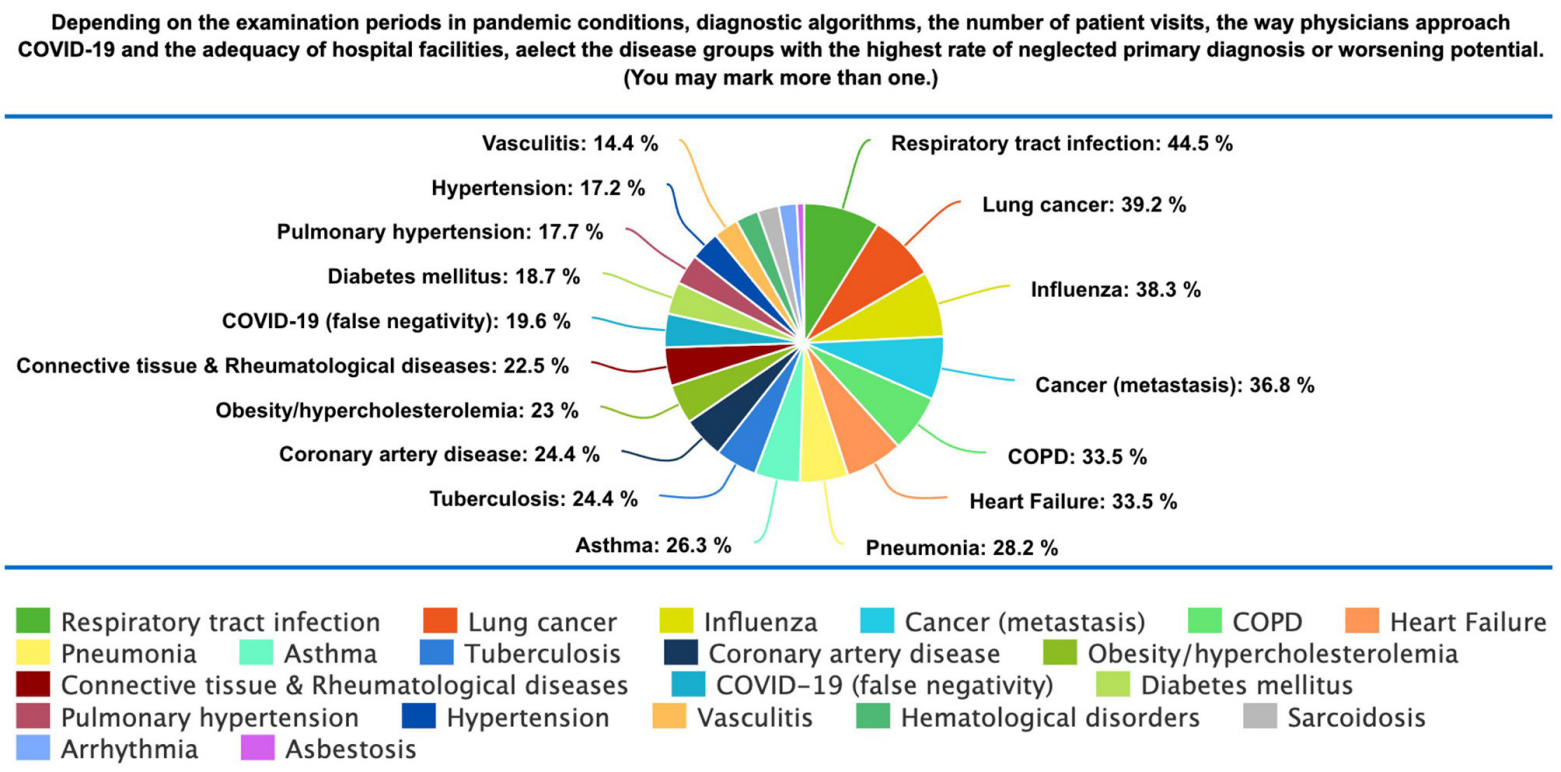
Distribution of insufficiently diagnosed diseases.

The distribution of delayed/neglected diagnosed diseases was given in Figure 1. The majority of HCPs reported that non-COVID-19 respiratory tract infections had the highest rate of neglected primary diagnosis, followed by lung cancer, Influenza, cancer (other), COPD, heart failure, and pneumonia.

### Factors Affecting Delayed and Neglected Diagnosis

The distribution and comparison of the factors that may cause a delay in the diagnosis of the patients were given in Supplementary Table 1. Among the HCPs who worked in the COVID-19 service, the ones who thought that there is a significantly increased negligence of the follow-up of various diseases and/or complication rates due to the COVID-19 pandemic(57.4%), were more likely to report that COVID-19 pandemic caused a delayed diagnosis of non-COVID-19 diseases compared to those who did not work in the COVID-19 service (*p* = 0.002). Although not significant, HCPs who thought that the suspicion or diagnosis of COVID-19 was less important in the patient profile applying to their outpatient clinic, more frequently claimed that COVID-19 occasionally caused delayed diagnosis of non-COVID-19 diseases.

The variables that may be associated with a COVID-19 centered clinical perspective were investigated in Supplementary Table 2. HCPs who stated that they have never dealt with a COVID-19 infected patient (66.7%), and who reported the ability to spend as much time with patients during the COVID-19 pandemic as before to believe HCPs were much more rigorous to diagnose or treat a COVID-19 case (or suspected COVID-19 case) than to diagnose/treat other serious diseases (*p* = 0.047). Although not significant, HCPs who thought that the workload intensity was higher after the pandemic (61.9%), tend to report an increased rate of being rigorous to diagnose COVID-19 (*p* = 0.095).

The relationship between telemedicine services and the answers to the questions about the follow-up of chronic diseases together are given in Supplementary Table 3. together with the potential causes of problems in the healthcare delivery. Although not significant, among those who think that the diagnosis and follow-up of chronic diseases are missed from time to time, and those who think that they are significantly affected, the number of HCPs who do not use telemedicine services was higher.

## Discussion

This paper includes a heterogeneous population of HCPs who continued their clinical practice during the COVID-19 pandemic in Turkey. The participants raised several concerns about the disruption of the health system which has had three consequences: an increase in delayed diagnoses, misdiagnoses, and neglected diseases. Secondary consequences of the disruptions should be questioned from the following perspectives: 1. What are the repercussions of disruptions? 2. Which departments are most affected? 3. Which diseases management has been most affected? 4. Are the hospital services adequate? 5. Are diagnostic and treatment procedures, which are delayed or not being met, the fault of the patient or is the onus on the healthcare provider?

### Impact of the COVID-19 Pandemic on Clinical Practice: Disease Diagnosis and Follow-Up

Most participants in our study felt that their workload intensity increased after the COVID-19 pandemic (42.1%). Although it seems that COVID-19 is primarily a respiratory disease that initially concerned pulmonologists, COVID-19 overwhelmed HCPs from all departments. Bennett et al. claim that pandemic practice is truly exceptional and requires collective and rapid action, thus Royal College of Physicians has maintained clinical advice and flowcharts for the non-pulmonology departments to effectively manage COVID-19 patients (13). During the COVID-19 pandemic, most HCPs were obligated to work in COVID-19 services due to the increased number of patients, either in outpatient clinics, inpatient services, and intensive care units in Turkey. Almost 70% of the participants reported that they worked in the COVID-19 service during the COVID-19 pandemic. These results are consistent with the observations by Bilaceraoglu, who reported that after March 2020, 26.5% of all hospital admissions were related to COVID-19 (14). El-Hage et al. underline that this situation significantly increased the workload and stress of HCPs from many departments including chest diseases, internal medicine, and cardiology, increasing their risk of undergoing burn-out, depression, or anxiety (15). HCPs who were overwhelmed by workload intensity may tend to have decreased time, attention, and quality of care for each patient, which may increase neglected diagnoses or misdiagnoses.

On the other hand, many surgeons had to cancel their elective operations due to hospitalization problems (lack of space, risk of infection), resulting in a decrease in their workload intensity, which may lead to an increase in work burden in the post-COVID era. This idea has been supported by a cohort study conducted by Bodilsen et al. for Danish hospital care (16). They also mentioned that during national lockdowns, the hospital admissions for significant musculoskeletal diseases and cancer reduced considerably along with the fewer number of incidental seek of care in the areas of pulmonology, cardiology, psychiatry, hematology, and gastroenterology.

Telemedicine is the use of telecommunication technology to provide remote healthcare to patients *via* telephone or video calls to seek virtual medical advice. It was reported that throughout April 2020, overall telehealth utilization for office visits and outpatient care was 78 times higher than in February 2020 in the United States (17). The view of telemedicine widely depends on the type of care delivered. According to Hincapie et al., telemedicine is an effective tool that has been increased its field of application with the pandemic, even though still problematic to be accessible, especially in rural areas (18). In Turkey, telemedicine services continue to spread out and get more convenient day by day. In our sample, 45.5% of the HCPs had never used telemedicine services. The high percentage of HCPs who never used telemedicine services in their practice seems to indicate that the difficulty of finding an appointment (54.1%; *p* = 0.032) caused a significant decrease in chronic care and routine screening, and a related increase in delayed diagnosis. More than half of the HCPs participated in this survey (54.5%) utilized telemedicine services during their clinical practice, however, they feel that its effectiveness both in first-time patients and follow-up patients was significantly low. It seems that the use of telemedicine services was not sufficient to tolerate the negative impact of COVID-19 on healthcare delivery.

One of the fundamental problems in the use of telemedicine is the inability to examine patients. A perfect diagnosis should stand from both a comprehensive medical history and a detailed physical examination. When a patient fails to report a key symptom that might have been noticeable during in-person care, it can compromise both diagnosis and treatment. In addition, Elawady et al. suggest that the inability to access patients' previous records stands as a handicap of the virtual system (19). However, telemedicine serves as a fair offer when there were barriers to patient care and treatment, such as during the lockdowns of the COVID-19 pandemic. Elawady et al. correspondingly demonstrate that 70% of the HCP agreed that telemedicine is beneficial to health care in their survey that was conducted in the United Kingdom. They also reported that the utilization of telemedicine by referring patients who need in-person care to clinics can result in increased quality of care for patients with chronic diseases. Elawady et al. concluded that using telemedicine may increase the rate of detection of diseases that require routine screening with lower costs, as long as the HCP is experienced and well-trained for the differentiation (19). Although its effectiveness was reported as low for many conditions and has the potential to result in neglect of many diagnoses depending on the users, offering telemedicine services is still important for those who are afraid to visit hospitals during COVID-19 pandemic due to the risk of infection and who have a hard time finding an appointment. Also, telemedicine can be a convenient option for conditions that do not require a physical examination or laboratory tests such as providing psychotherapy. Further studies are required on why HCPs do not find telemedicine to be effective, and further training programs should be arranged on the use of telemedicine to improve its effectiveness and extensiveness.

### Factors Affecting Delayed and Neglected Diagnosis

In general, HCPs were not completely satisfied with the time they spared for each patient and disease spectrums after COVID-19 pandemic restrictions were lifted. Almost half of the HCPs stated that they consider COVID-19 as a priority diagnosis in the patients applying to clinics. This resulted in nearly 60% of HCPs reporting a decrease in the variety of cases they see on the monthly basis, and 10% of HCPs reporting only dealing with COVID-19 patients. Some physicians (18.2%), if not all, reported that COVID-19 is 81–100% important in their daily clinical perspective. In addition, more than 80% of HCPs reported that they had patients who were not correctly diagnosed since the wide spectrum of clinical presentation of COVID-19 predominates the diagnostic process, even when their COVID-19 tests were negative. It seems that 1 out of 2 people had a delayed or misdiagnosis. Today, many case reports refer to the occasions where COVID-19 pneumonia is taken into account instead of drug-induced pneumonitis, granulomatous polyangiitis, or cytomegalovirus infection, as examples (20–22).

HCPs reported that the laboratory and radiological tests requested for diagnosis/follow-up of patients were significantly increased compared to the pre-pandemic era. This creates a paradox, since further testing may encourage HCPs to face unconsidered diagnoses. The critical point in this finding is that laboratory and radiological findings include SARS-CoV-2 RNA or antigen detection and chest x-rays. However, these techniques are not diagnostic for many diseases. On the other hand, bronchoalveolar lavage, bronchoscopy, sputum cultures, and many respiratory disease-related specific tests are expected to be lower in number during the pandemic. To overcome this issue, many guidelines have been published to differentiate radiological or laboratory presentations of COVID-19 from similar diseases. For instance, WHO released an additional guideline to differentiate tuberculosis from COVID-19 clinically (23).

Real-time-polymerase chain reaction (RT-PCR) tests are among the primary laboratory tests for COVID-19. A meta-analysis revealed that false-negative result rates of RT-PCR were reported as ranging between 20–66%, depending on the days from symptoms onset and viral load (24). Pecoraro et al. estimated that a range between 2–58/1,000 subjects could be misdiagnosed with a disease prevalence of 10%, increasing to 290/1,000 misdiagnosed subjects with a disease prevalence of 50% with false-negative results. They also reported that up to 58% of COVID-19 patients may have initial false-negative RT-PCR results (25). On the other hand, Dinnes et al. provide that a hypothetical cohort of 1,000 participants who has been suspected of COVID-19 resulted in only 105 positive test results, while 10 patients had false-positive results. In the same sample, they underline that within 895 negative results, only 5 of them ended up being false negatives (26). This data significantly justifies the need to implement a correct diagnostic strategy to precisely identify suspected cases by combining analytical quality with more involvement in diagnostic-therapeutic pathways. HCPs perceived that the majority of overlooked diseases belonged to lung diseases, followed by infectious diseases, heart diseases, cancer, and local manifestations of other systemic diseases, consistent with the results in other studies (27, 28).

Although an information bias exists in this perspective since it's hard for doctors to admit their negligence and it's easy to blame disrupted healthcare services and patients' negligence, HCPs increasingly think that there exists an increase in negligence in various diseases and complication rates during COVID-19 pandemic. Sevinc et al. reported that the presentation of non-COVID pulmonary pathologies such as COPD and ILD has decreased significantly, and there was a change in the profile of the patients, mainly presenting with asthma, pneumonia, and pulmonary thromboembolism, which might all result from COVID-19 related complications (29).

The majority of the HCPs reported that vaccination and normalization significantly facilitate the delivery of healthcare, the application of patients to clinics, and the increase in non-COVID-19 case diversity. It is worthy of note that some HCPs reported that the case diversity could not reach the pre-pandemic era, and there were still patients who did not apply to physicians even after normalization. This may have several reasons including the continuing fear of COVID-19, or refusing the COVID-19 vaccines (27). Overall, the consensus about vaccination is summarized by Moghadas et al. that the vaccination significantly revived healthcare services in the US, which had been interrupted for a while, by reducing the number of cases, hospitalization, and deaths due to COVID-19 with the mitigation of outbreaks, yielding the floor to other important reasons of healthcare (30).

COVID-19 pandemic has placed a huge and indefinite burden on healthcare professionals and left them in huge psychological distress (31). COVID-19 pandemic also caused a significant impact on the patient-physician communication and mental health of the physicians. 4 out of 10 HCPs reported a more negative/problematic communication than before the pandemic, while 8 out of 10 participants reported that the mental health of physicians was adversely affected. HCPs also reported a significant amount of fear of being infected with SARS-CoV-2 and even more fear of infecting their family and patients. This may significantly impact the time spared for each patient, particularly when there was a suspicion of COVID-19. Although most HCPs think that hospital services and personal protective equipments provided by their hospital are nearly sufficient, the risk of transmitting COVID-19 significantly increases with increased viral load and contact with the patient using physical examination and detailed medical history. It was reported that HCPs are at increased risk of COVID-19 (32), consistent with 40% of the participants having been infected with SARS-CoV-2 during the pandemic. As a result of a pandemic that takes so much space on the agenda and the disease that presents so vaguely in a variety of different pictures, more than 60% of the HCPs think that physicians are being much more rigorous to diagnose COVID-19. Feelings of being afraid, demoralized, and overwhelmed significantly affect the decision process for many diagnoses (33).

Most of the participants did not consider hospital facilities, the number of appointments, and healthcare services as completely sufficient, causing a huge interrupted healthcare services burden. This is also consistent with previous studies reporting a universal decrease in healthcare utilization for non-COVID-19 conditions, across both high and lower-income countries (34). HCPs in this study indicated a significant negative impact of COVID-19 in detection, management, and ongoing support for patients with existing chronic conditions. Most of them arose from the inability/hesitation of visiting hospitals during the pandemic due to the higher risk of infection. Other reasons include the difficulty of finding an appointment due to the increased burden on healthcare services and automatic extension of medication reports for chronic diseases. Singh et al. implied that COVID-19 not only causes harm to the communities by direct infection but also results in deterioration in public health by missing out on previously known diseases such as diabetes (35). They demonstrated that out of 5,672 people with chronic diseases, taken from 5 different countries, participants had increased burden of deteriorated economic status, and reported that getting care became harder after the COVID-19 pandemic. The diabetes symptoms worsened for some of the patients throughout the course of the pandemic. Reports suggest that perceived less urgent aspects of care, such as routine screening and chronic disease management have been left undergone during the COVID-19 pandemic (36, 37). Healthcare authorities and policymakers should create a strategy that will support early intervention to address risk factors and conditions that are likely to result in increased morbidity and mortality in such populations. Populations that have limited access to healthcare due to low socioeconomic status and are at risk of increased complications of COVID-19 should particularly be considered for preventative healthcare. These findings highlight a need for urgent work in this area to inform workforce strategy and capacity building.

In the optional section, which consists of a single open-ended question, some survey participants anonymously emphasized that the diseases in the post-pandemic world are distributed in two piles in clinical practice: the COVID-19 and the others; where the RT-PCR negative cases strain to attain the diagnosis and treatment due to fear of contamination, impaired communication, increased workload of pulmonologists and nonappointment. It is no doubt that the COVID-19 pandemic affected the professional self-confidence of medical doctors due to its catastrophic, vague, and abrupt emergence. It altered the traffic in health care facilities, exploiting the effort and consideration of HCP. The prevention, control, and treatment of all diseases, not only COVID-19, should be included in the health system of all countries, even at extraordinary time intervals. We anticipate that this atmosphere ceases as soon as possible, enabling broader management of public health problems in Turkey.

The strength of this study is the fact that it is conducted in widespread health facilities and included many types of departments. The opinions of participants were asked deeply and a large spectrum of topics was covered including the whole process of pandemic. Both private and state hospitals were included in the study that bringing a broader understanding of the health system in Turkey. It is found that the parameters in this study, which were focused on the diagnosis and follow-up interruptions in the Turkish health care system after the pandemic, were least affected by the gender or the medical department of the participants, excluding the suspicion that certain gender physicians or certain specialties are more sensitive in emergency conditions. Hence, findings from this study can be implemented in many aspects of the health system, regardless of the personal characteristics or the medical environment of the physician. We hope that findings from this study will reach the healthcare authorities and contribute to policymaking to enhance the quality of care in Turkey.

This survey was conducted during a rise in COVID-19 cases due to decreased protection of vaccines and the lack of patients receiving boosters in Turkey. This likely impacted the sample size. Despite this limitation, the sample size in this study is comparable with that of other research in this participant group. This study did not observe significant differences in the effects of the COVID-19 pandemic between different departments. These findings require further investigation in larger studies following the pandemic to further explore findings. The sources of bias can be associated with the genuine response honesty and snowball sampling, where the most sensitive and upfront medical doctors on this topic choose to participate in this survey, along with the subjectiveness of the physicians' self-awareness for a “late or missed diagnoses.” Regarding this topic, we support possible future studies which aim to correlate HCPs' perceptions with objective data from hospitals or a national database.

## Conclusion

This is the first survey of healthcare providers evaluating the impact of COVID on healthcare services in Turkey. Policymaking requires this evidence to drive changes to better support HCPs in Turkey. Understanding the perceptions of HCPs and their experiences during a pandemic is fundamental to the ongoing optimization of healthcare and support. This study highlights the impact COVID-19 has had on the healthcare services in Turkey through the eyes of medical doctors. Findings from this study emphasize that HCPs perceive that COVID-19 has significantly caused delayed diagnoses and interruption in the management of chronic diseases, and also increased the risk of missing out on non-COVID-19 diseases which either show similar signs and symptoms as COVID-19 or could not be diagnosed due to pandemic circumstances hindering the access of first-time and follow-up patients.

## Data Availability Statement

The raw data supporting the conclusions of this article will be made available by the authors, upon reasonable request.

## Ethics Statement

This study was approved by the Republic of Turkey Ministry of Health and Ondokuz Mayis University Institutional Review Board (No: 2021/599) with respect to its scientific content. The patients/participants provided their written informed consent to participate in this study.

## Author Contributions

All authors have agreed on the final version and contributed equally to the presented work.

## Conflict of Interest

The authors declare that the research was conducted in the absence of any commercial or financial relationships that could be construed as a potential conflict of interest.

## Publisher's Note

All claims expressed in this article are solely those of the authors and do not necessarily represent those of their affiliated organizations, or those of the publisher, the editors and the reviewers. Any product that may be evaluated in this article, or claim that may be made by its manufacturer, is not guaranteed or endorsed by the publisher.
