# Supplementary material for: A Glance Into Healthcare Delivery During COVID-19 Pandemic: A Survey Among Turkish Medical Doctors
Source: Front Med (Lausanne). 2022 Jul 19;9:890417. doi: 10.3389/fmed.2022.890417 (PMC9345501; doi:10.3389/fmed.2022.890417)
Supplement: Supplementary file 1 [file Table_1.DOCX]

**Online Survey : Impact of the COVID-19 Pandemic on General Health Services**

Dear Participant,

This questionnaire is made to be used in the scientific research named “The Effect of the COVID-19 Pandemic on General Health Services, Patient Diagnosis and Follow-up Processes”. The information you provide will be used for research purposes only and does not contain your identifying personal information (name, surname, etc.). This survey will take approximately 10 minutes to complete. We request you to answer all questions sincerely. Thank you for your support of our scientific work.

Communication: Principal Investigator-Professor Dr. Sevket Ozkaya, VM Medicalpark Samsun Hospital, Chest Diseases Clinic. [ozkayasevket@yahoo.com](mailto:ozkayasevket@yahoo.com) +90 532 474 13 09

**Do you agree to participate in this survey and have your answer published anonymously?**

I agree. (This is a mandatory answer to participate.)

**I. Demographic Information:**

1. Age
2. Gender
3. Woman
4. Male
5. I prefer not to specify.
6. Academic Title and Department
7. Professional experience (in years)
8. Average number of patients you encounter with per day
9. Have you worked in the COVID-19 service during the pandemic?
10. Yes
11. No
12. Have you had COVID-19?
13. Yes
14. No

**II. Impact of the COVID-19 Pandemic on Clinical Practice**

1. The medical unit you are in charge of
2. Public Hospital
3. Private Hospital
4. Personal Clinic
5. Other: (please specify)
6. The main area of work
7. Outpatient clinic
8. Inpatient clinic/Service
9. Intensive care unit
10. Operating room
11. Other: (please specify)
12. Please compare the current workload density in your outpatient clinic/emergency/medical unit you with the pre-pandemic era (before March 2020).
13. More intense before the pandemic
14. Similar or equal
15. More intense after the pandemic
16. Can you spare as much time as before the pandemic for the patients who had different kinds of diseases other than COVID-19 after the restrictions were lifted? (1: Certainly not, 10: Certainly yes) (median,min-max)(mean$\pm SD$)

Certainly Not **1 2 3 4 5 6 7 8 9 10** Certainly Yes

1. On average, how important is the suspicion or diagnosis of COVID-19 (meaning PCR requirement, positivity or radiological/clinical findings) by percentage (%) in the patient profile applying to outpatient clinic that you encounter in your daily practice?
2. 0-20
3. 21-40
4. 41-60
5. 61-80
6. 81-100
7. Considering the normalisation period started after vaccination, evaluate the monthly non-COVID-19 case diversity between 1 and 10 by comparing it to the pre-pandemic period (before March 2020).

Only COVID-19, no diversity **1 2 3 4 5 6 7 8 9 10** Maximum diversity, similar to pre-pandemic

1. Evaluate your monthly non-COVID-19 case diversity by comparing the time before March 2020 with the peak period of the pandemic.
2. During the peak period of the pandemic, I encountered a wide variety of cases, other than COVID-19.
3. During the peak period of the pandemic, similar or the same variety of cases applied as in the pre-pandemic period.
4. During the peak period of the pandemic, I encountered a small variety of cases, other than COVID-19.
5. During the peak of the pandemic, I only dealt with COVID-19 patients.
6. During the peak of the pandemic, I have not dealt with any COVID-19 patients.
7. Does your institution/medical unit use telemedicine services in its routine clinical procedures?
8. Mostly
9. Sometimes
10. Never
11. If you answered "mostly" or "sometimes" to the previous question, rate the effectiveness of teleclinics for first-time patients. (If you answered "never", your answer in this question will not be considered.)

Not Effective **1 2 3 4 5 6 7 8 9 10** Highly Effective

1. If you answered "mostly" or "sometimes" two questions before, rate the effectiveness of teleclinics in follow-up patients. (If you answered "never", your answer in this question will not be considered.)

Not Effective **1 2 3 4 5 6 7 8 9 10** Highly Effective

**Iii. The Effect of COVID-19 Pandemic on Diagnosis and Follow-up**

1. Evaluate the compliance of patients who require chronic and/or routine screening (cancer, diabetes, hypertension, asthma, COPD, etc.) from March 2020 to the present, to apply to the hospital or to comply with the controls, on a scale of 1 to 10, according to the majority. (1:No control visits 10: All controls have been done without interruption) (median,min-max)(mean$\pm SD$)

No control visits:**1 2 3 4 5 6 7 8 9 10:**All controls have been done without interruption

1. Which of the following is most likely to be the reason for your answer to the previous question? (You can mark more than one reason.)
2. Difficulty of finding an appointment
3. Automatic extension of medication reports
4. Idea that health-care centers carry a higher risk of COVID-19
5. Patient's personal neglect (similar or same as before the pandemic)
6. Unability or hesitation to visit hospitals during the pandemic
7. Financial reasons
8. Other: (please specify)
9. Did you encounter any problems in the health services of non-COVID-19 diseases throughout the pandemic? (You can mark more than one.)
10. Difficulty of finding medications
11. Lack of control/follow-up
12. Increase in late diagnoses
13. Increasing misdiagnoses
14. Affected surgical decisions
15. Hospitalization problems (lack of space, risk of infection, etc.)
16. Healthcare services in areas other than COVID-19 are the same as before March 2020.
17. Health services in areas other than COVID-19 are better than before March 2020.
18. Other: (please specify)
19. Have you had any cases who were mistakenly diagnosed in the first admission since the suspicion of a possible COVID-19 infection in the patient predominates the diagnostic process (eg. not excluding COVID-19 even though the PCR test is negative)?
20. Always
21. Often
22. Sometimes
23. Rarely
24. Never
25. If you answered "always", "often", "sometimes" or "rarely" to the previous question, which of the following is most appropriate for the interdisciplinary distribution of these diseases?
26. Chest diseases
27. Heart diseases
28. Metabolic diseases
29. Neurological diseases
30. Infectious diseases
31. Cancers
32. Local manifestations of systemic diseases
33. Other: (please specify)
34. Evaluate the patient-physician communication by comparing it with the pre-pandemic era (before March 2020).
35. More positive/effective communication than before the pandemic
36. Similar or same communication as before the pandemic
37. More negative/problematic communication than before the pandemic
38. Do you think that there is an increase in the follow-up of various diseases and/or complication rates due to the negligence of the COVID-19 pandemic? Please score between 1 and 10.

Absolutely Not **1 2 3 4 5 6 7 8 9 10** Absolutely Yes

1. Do you think there is an increase in the rate of laboratory and radiological tests required for diagnosis/follow-up of patients after normalization, compared to pre-pandemic (March 2020)?

Certainly Not **1 2 3 4 5 6 7 8 9 10** Certainly Yes

1. Do you think that after the normalization, the clinical services, the number of appointments and the hospital facilities are sufficient for the diagnosis and follow-up of the patients before the pandemic (March 2020)?

Certainly Not **1 2 3 4 5 6 7 8 9 10** Certainly Yes

1. Do you think that health-care professionals are being much more rigorous to diagnose/treat an uncertain disease like COVID-19 than to other important diseases?
2. Yes
3. No
4. I don't have an opinion.
5. Do you think that the diagnosis of diseases whose symptoms are similar to COVID-19 may have been neglected due to the timeliness of COVID-19? (Respondents are allowed to choose more than one answer since the choices include 3 scenerios for chronic diseases and 3 for COVID-19 mimicking ones.)
6. I think that the diagnosis and follow-up of chronic diseases are mostly missed.
7. I think that the diagnosis and follow-up of chronic diseases are missed from time to time.
8. I do not think that the diagnosis and follow-up of chronic diseases are significantly affected.
9. I think that some diseases that show similar signs and symptoms as COVID-19 are mostly not diagnosed correctly.
10. I think that some diseases that show similar signs and symptoms with COVID-19 are not properly diagnosed from time to time.
11. I do not think that the diagnosis of diseases with similar signs and symptoms as COVID-19 is affected.
12. Other: (please specify)
13. Depending on the examination periods in pandemic conditions, diagnostic algorithms, the number of patient visits, the way physicians approach COVID-19 and the adequacy of hospital facilities, aelect the disease groups with the highest rate of neglected primary diagnosis or worsening potential. (You can mark more than one.)
14. Covid-19
15. Influenza
16. Respiratory tract infections (other)
17. Lung cancer
18. Cancer (other) and metastasis
19. COPD
20. Asthma
21. Pneumonia
22. Tuberculosis
23. Sarcoidosis
24. Vasculitis
25. Asbestos
26. Diabetes
27. Hypertension
28. Arrhythmia
29. Heart failure
30. Coronary Artery Disease
31. Pulmonary hypertension
32. Connective tissue diseases / rheumatological diseases
33. Hematological disorders
34. Metabolic disorders (obesity, high cholesterol, etc.)

**IV. Rating of Feelings and Fears**

1. Are you afraid of being infected with SARS-CoV-2?

Not at all **1 2 3 4 5** Definitely yes

1. Do you think that the hospital services and personal protective equipment provided by your hospital are sufficient?

Not at all **1 2 3 4 5** Definitely yes

1. Are you afraid of infecting your family and patients with SARS-CoV-2?

Not at all **1 2 3 4 5** Definitely yes

1. With vaccination and normalization, has there been an increase in appointments for non-COVID-19 diseases?
2. Yes
3. No
4. I don't have an opinion.
5. Do you think that vaccination facilitates the patient diagnosis and follow-up processes during the COVID-19 pandemic?

Not at all **1 2 3 4 5** Definitely yes

1. How do you think a COVID-19-centered clinical practice, which is implemented during the pandemic, affects field physicians mentally during their diagnostic processes?
2. The mental health of physicians were adversely affected.
3. The mental health of physicians were affected positively.
4. The mental health of physicians were not affected.
5. I don't have an opinion.
6. Other: (please specify)

**You have reached the end of our survey. Thank you for your participation.**

Optional comments:
