# Supplementary material for: A Glance Into Healthcare Delivery During COVID-19 Pandemic: A Survey Among Turkish Medical Doctors
Source: Front Med (Lausanne). 2022 Jul 19;9:890417. doi: 10.3389/fmed.2022.890417 (PMC9345501; doi:10.3389/fmed.2022.890417)
Supplement: Supplementary file 2 [file Data_Sheet_1.docx]

**SUPPLEMENTARY**

**Table 1. Impact of the COVID-19 predominance in general healthcare and its related factors**

| **Variables** | | Have you had any cases who were **mistakenly diagnosed in the first admission** since the suspicion **of a possible COVID-19 infection in the patient predominates the diagnostic process (eg. not excluding COVID-19 even though the PCR test is negative)?** | | | | |  |
| --- | --- | --- | --- | --- | --- | --- | --- |
|  |  | **Very Often** | **Sometimes** | **Rarely** | **Never** | **P** | |
| Years of professional experience | | 13 (1-30) | 18 (1-45) | 14.5 (1-40) | 15 (1-55) | 0.415^a^ | |
| Average number of patients that you encounter per day | | 40 (7-100) | 35 (5-129) | 40 (3-1200) | 30 (2-1200) | 0.350^a^ | |
| HCPs who had COVID-19 | Yes | 7 (8.2%) | 36 (42.4%) | 30 (35.3%) | 12 (14.1%) | 0.529^b^ | |
|  | No | 10 (8.1%) | 57 (46%) | 33 (26.6%) | 24 (19.4%) |  |  |
| HCPs afraid of being infected with SARS-CoV-2 (1: Not at all, 5: Definitely yes) | | 4 (1-5) | 4 (1-5) | 4 (1-5) | 4 (1-5) | 0.209^a^ | |
| HCPs afraid of infecting their family with SARS-CoV-2 (1: Not at all, 5: Definitely yes) | | 5 (1-5) | 5 (1-5) | 5 (1-5) | 5 (1-5) | 0.199^a^ | |
| Monthly case diversity | Few diverse cases | 11 (8.9%) | 54 (43.5%) | 42 (33.9%) | 17 (13.7%) | **0.009^b^*** | |
|  | Wide variety of cases | 0 (0%) | 9 (64.3%) | 3 (21.4%) | 2 (14.3%) |  |  |
|  | I've never dealt with COVID-19 patients | 0 (0%) | 5 (41.7%) | 1 (8.3%) | 6 (50%) |  |  |
|  | Similar or same kind of case diversity as pre-pandemic | 1 (2.9%) | 13 (37.1%) | 11 (31.4%) | 10 (28.6%) |  |  |
|  | I’ve only dealt with COVID-19 patients | 5 (20.8%) | 12 (50%) | 6 (25%) | 1 (4.2%) |  |  |
| Can you spare as much time as before the pandemic for the patients who had different kinds of diseases other than COVID-19 after the restrictions were lifted? (1: Certainly not, 10: Certainly yes) (median,min-max)(mean$\pm SD$) | | 6 (1-10) | 6 (1-10) | 5 (1-10) | 6.5 (1-10) | 0.762^a^ | |
| Gender | Female | 8 (7%) | 46 (40.4%) | 38 (33.3%) | 22 (19.3%) | 0.419^b^ | |
|  | Male | 9 (9.5%) | 47 (49.5%) | 25 (26.3%) | 14 (14.7%) |  |  |
| Academic title | Research associate | 0 (0%) | 4 (57.1%) | 2 (28.6%) | 1 (14.3%) | 0.928^b^ | |
|  | Resident doctor | 2 (4.8%) | 19 (45.2%) | 14 (33.3%) | 7 (16.7%) |  |  |
|  | Attending doctor | 6 (10.2%) | 30 (50.8%) | 16 (27.1%) | 7 (11.9%) |  |  |
|  | Associate professor | 1 (5%) | 9 (45%) | 6 (30%) | 4 (20%) |  |  |
|  | Professor | 0 (0%) | 5 (38.5%) | 4 (30.8%) | 4 (30.8%) |  |  |
|  | General practitioner | 1 (9.1%) | 4 (36.4%) | 4 (36.4%) | 2 (18.2%) |  |  |
|  | Assistant professor | 3 (15%) | 9 (45%) | 3 (15%) | 5 (25%) |  |  |
| Department | Departments that encounter COVID-19 as a first line(chest diseases, infectious diseases, ICU, emergency medicine, internal medicine) | 11 (13.1%) | 37(44%) | 27(46.6%) | 9 (10.7%) | 0.112^b^ | |
|  | Other internal sciences | 2 (4%) | 15 (30%) | 19 (38%) | 14 (28%) |  |  |
|  | Surgical sciences | 3 (7.9%) | 16 (42.1%) | 12 (31.6%) | 7 (18.4%) |  |  |
| Have you worked in the COVID-19 service during the pandemic? | Yes | 16 (11.2%) | 66 (46.2%) | 45 (31.5%) | 16 (11.2%) | **0.002^b^*** | |
|  | No | 1 (1.5%) | 27 (40.9%) | 18 (27.3%) | 20 (30.3%) |  |  |
| Medical unit | Family health center | 1 (7.1%) | 5 (35.7%) | 6 (42.9%) | 2 (14.3%) | 0.860^b^ | |
|  | Public hospital | 9 (8.1%) | 49 (44.1%) | 34 (30.6%) | 19 (17.1%) |  |  |
|  | Private hospital | 3 (5.5%) | 26 (47.3%) | 14 (25.5%) | 12 (21.8%) |  |  |
|  | Training and research hospitals/ University hospitals | 4 (13.8%) | 13 (44.8%) | 9 (31%) | 3 (10.3%) |  |  |
| The main area of work | Outpatient clinic | 11 (7.7%) | 60 (42%) | 45 (31.5%) | 27 (18.9%) | 0.582^b^ | |
|  | Intensive care unit | 3 (12.5%) | 14 (58.3%) | 6 (25%) | 1 (4.2%) |  |  |
|  | Inpatient clinic/Service | 2 (6.7%) | 15 (50%) | 9 (30%) | 4 (13.3%) |  |  |
|  | Operating room | 1 (8.3%) | 4 (33.3%) | 3 (25%) | 4 (33.3%) |  |  |
| Comparison of the the current workload intensity in the outpatient clinic/emergency room/medical unit you are assigned with with the pre-COVID-19 pandemic era (before March 2020) | Similar or equal | 5 (6.3%) | 36 (45.6%) | 23 (29.1%) | 15 (19%) | 0.893^b^ | |
|  | More intense before the pandemic | 4 (9.5%) | 18 (42.9%) | 11(26.2%) | 9 (21.4%) |  |  |
|  | More intense after the pandemic | 8 (9.1%) | 39 (44.3%) | 29 (33%) | 12 (13.6%) |  |  |
| On average, how important is the suspicion or diagnosis of COVID-19 (meaning PCR requirement, positivity or radiological/clinical findings) by percentage (%) in the patient profile applying to outpatient clinic that you encounter in your daily practice? | 0-20% | 4 (9.3%) | 17 (39.5%) | 9 (20.9%) | 13 (30.2%) | 0.071^b^ | |
|  | 21-40% | 1 (2.9%) | 15 (42.9%) | 12 (34.3%) | 7 (20%) |  |  |
|  | 41-60% | 6 (14%) | 15 (34.9%) | 15 (34.9%) | 7(16.3%) |  |  |
|  | 61-80% | 4 (8%) | 31 (62%) | 12 (24%) | 3 (6%) |  |  |
|  | 81-100% | 2 (5.3%) | 15 (39.5%) | 15 (39.5%) | 6(15.8%) |  |  |
| Considering the normalisation period started after vaccination, evaluate the monthly non-COVID-19 case diversity between 1 and 10 by comparing it to the pre-pandemic period (before March 2020). 1:Only COVID-19, 10:Wide variety of cases | | 7 (2-10) | 8 (1-10) | 7 (1-10) | 7.5 (3-10) | 0.839^a^ | |
| Do you think that vaccination facilitates the patient diagnosis and follow-up processes during the COVID-19 pandemic? 1:not at all, 5:definitely yes | | 4 (1-5) | 4 (1-5) | 5 (1-5) | 5 (1-5) | 0.071^a^ | |
| With vaccination and normalization, has there been an increase in **appointments** for non-COVID-19 **diseases**? | Yes | 9 (6.1%) | 65 (43.9%) | 45 (30.4%) | 29 (19.6%) | 0.462^b^ | |
|  | No | 5 (14.3%) | 15 (42.9%) | 12(34.3%) | 3 (8.6%) |  |  |
| Do you think that there is an increase negligence of the follow-up of various diseases and/or complication rates due to COVID-19 pandemic? Score between 1 and 10. (1:Absolutely no 10:Absolutely yes) | | 8 (3-10) | 7 (1-10) | 6 (1-10) | 6 (1-10) | **0.001^a^*** | |
| Do you think that there is an increase in the rate of laboratory and radiological tests required for diagnosis/follow-up of patients after normalization compared to pre-pandemic (March 2020)? (0:Certainly not, 10:Certainly yes) | | 7 (1-10) | 7 (2-10) | 8 (1-10) | 7 (1-10) | 0.472^a^ | |
| Do you think that after the normalization, the clinical services, the number of appointments and the hospital facilities are sufficient for the diagnosis and follow-up of the patients before the pandemic (March 2020)? (0:Certainly not, 10 Certainly yes) | | 5 (1-10) | 5 (1-10) | 6 (1-10) | 6.5 (2-10) | 0.410^a^ | |
| Evaluate the patient-physician communication by comparing it with the pre-pandemic (March 2020). | More positive communication than before the pandemic | 2 (7.4%) | 12 (44.4%) | 10 (37%) | 3 (11.1%) | 0.429^b^ | |
|  | More negative/problematic communication than before the pandemic | 7 (8.8%) | 39 (48.8%) | 25 (31.3%) | 9 (11.3%) |  |  |
|  | Similar or same communication as before the pandemic | 8 (7.8%) | 42 (41.2%) | 28 (27.5%) | 24 (23.5%) |  |  |
| Evaluate the **compliance** of patients who require chronic and/or routine screening (cancer, diabetes, hypertension, asthma, COPD, etc.) from March 2020 to the present, to apply to the hospital or to comply with the controls, on a scale of 1 to 10, according to the majority. (1:No control visits 10: All controls have been done without interruption) (median,min-max)(mean$\pm SD$) | | 6 (1-8) | 4 (1-9) | 4 (2-9) | 5 (1-9) | 0.438^a^ | |
| Do you think that the hospital services and personal protective equipment provided by your hospital are sufficient? (1: Not at all, 5:Definitely yes) | | 4 (1-5) | 4 (1-5) | 3 (1-5) | 4 (1-5) | 0.579^a^ | |
| How do you think a COVID-19-centered clinical practice, which is implemented during the pandemic, affects field physicians mentally during their diagnostic processes? | The mental health of physicians were not affected. | 1 (12.5%) | 5 (62.5%) | 2 (25%) | 0 (0%) | 0.132^b^ | |
|  | The mental health of physicians were affected positively. | 1 (8.3%) | 7 (58.3%) | 2 (16.7%) | 2 (16.7%) |  |  |
|  | The mental health of physicians were adversely affected. | 14 (8.2%) | 75 (43.9%) | 56 (32.7%) | 26 (15.2%) |  |  |

a:Kruskall-Wallis h test;b: Chi-Square Test;*:p<0.01

Numeric variables that are not normally distributed are median (min-max); categorical variables are presented as numbers (%lines)

**Table 2. Effect of the COVID-19-centered clinical perspective on other parameters**

| **Variables** | | **Do you think that health-care professionals are being much more rigorous to diagnose/treat an uncertain disease like COVID-19 than to other important diseases?** | | | |
| --- | --- | --- | --- | --- | --- |
|  |  | **Yes** | **No** | **No opinion** | **P** |
| Years of professional experience | | 15 (1-55) | 16 (1-40) | 20.5 (1-43) | 0.518^a^ |
| Average number of patients that you encounter per day | | 35 (3-1200) | 45 (2-200) | 30 (10-1200) | 0.329^a^ |
| HCPs who had COVID-19 | Yes | 51 (60%) | 25 (29.4%) | 9 (10.6%) | 0,457^b^ |
|  | No | 76 (61.5%) | 29 (23.4%) | 19 (15.3%) |  |
| HCPs afraid of being infected with SARS-CoV-2 (1: Not at all, 5: Definitely yes) | | 4 (1-5) | 4 (1-5) | 4 (1-5) | 0.679^a^ |
| HCPs afraid of infecting their family with SARS-CoV-2 (1: Not at all, 5: Definitely yes) | | 5 (1-5) | 5 (1-5) | 5 (1-5) | 0.371^a^ |
| Monthly case diversity | Few diverse cases | 78 (62.9%) | 31 (25%) | 15 (12.1%) | **0.047^b**^** |
|  | Wide variety of cases | 5 (35.7%) | 5 (35.7%) | 4 (28.6%) |  |
|  | I've never dealt with COVID-19 patients | 8 (66.7%) | 0 (0%) | 4 (33.3%) |  |
|  | Similar or same kind of case diversity as pre-pandemic | 24 (68.6%) | 8 (22.9%) | 3 (8.6%) |  |
|  | I’ve only dealt with COVID-19 patients | 12 (50%) | 10 (41.7%) | 2 (8.3%) |  |
| Can you spare as much time as before the pandemic for the patients who had different kinds of diseases other than COVID-19 after the restrictions were lifted? (1: Certainly not, 10: Certainly yes) (median,min-max)(mean$\pm SD$) | | 7 (1-10) | 5 (1-10) | 6 (2-10) | **0.013^a**^** |
| Gender | Female | 69 (60.5%) | 30 (26.3%) | 15 (13.2%) | 0.983^b^ |
|  | Male | 58 (61.1%) | 24 (25.3%) | 13 (13.7%) |  |
| Academic title | Research assistant | 5 (71.4%) | 1(14.3%) | 1(14.3%) | 0.108^b^ |
|  | Resident doctor | 24 (57.1%) | 12 (28.6%) | 6 (14.3%) |  |
|  | Attending doctor | 36 (61%) | 19 (32.2%) | 4 (6.8%) |  |
|  | Associate professor | 13 (65%) | 5 (25%) | 2 (10%) |  |
|  | Professor | 7 (53.8%) | 2 (15.4%) | 4 (30.8%) |  |
|  | General practitioner | 7 (63.6%) | 0 (0%) | 4 (36.4%) |  |
|  | Assistant professor | 16 (80%) | 3 (15%) | 1 (5%) |  |
| Department | Departments that encounter COVID-19 as a first line(chest diseases, infectious diseases,ICU emergency medicine, internal medicine) | 50 (59.5%) | 22 (26.2%) | 12 (14.3%) | 0.987^b^ |
|  | Other internal sciences | 31 (62%) | 11 (22%) | 8 (16%) |  |
|  | Surgical sciences | 23 (60.5%) | 9 (23.7%) | 6 (15.8%) |  |
| Have you worked in the COVID-19 service during the pandemic? | Yes | 86 (60.1%) | 40 (28%) | 17 (11.9%) | 0.450^b^ |
|  | No | 41 (62.1%) | 14 (21.2%) | 11 (16.7%) |  |
| Medical unit | Family health center | 5 (35.7%) | 7 (50%) | 2 (14.3%) | 0.125^b^ |
|  | Public hospital | 63 (56.8%) | 33 (29.7%) | 15 (13.5%) |  |
|  | Private hospital | 40 (72.7%) | 8 (14.5%) | 7 (12.7%) |  |
|  | Training and research hospitals/ University hospitals | 19 (65.5%) | 6 (20.7%) | 4(13.8%) |  |
| The main area of work | Outpatient clinic | 90 (62.9%) | 36 (25.2%) | 17 (11.9%) | 0.246^b^ |
|  | Intensive care unit | 13 (54.2%) | 8 (33.3%) | 3 (12.5%) |  |
|  | Inpatient clinic/Service | 14 (46.7%) | 10 (33.3%) | 6 (20%) |  |
|  | Operating room | 10 (83.3%) | 0 (0%) | 2 (16.7%) |  |
| Comparison of the the current workload intensity in the outpatient clinic/emergency room/medical unit you are assigned with with the pre-COVID-19 pandemic era (before March 2020) | Similar or equal | 50 (63.3%) | 20 (25.3%) | 9 (11.4%) | 0.095^b^ |
|  | More intense before the pandemic | 26 (61.9%) | 6 (14.3%) | 10 (23.8%) |  |
|  | More intense after the pandemic | 51 (58%) | 28 (31.8%) | 9 (10.2%) |  |
| On average, how important is the suspicion or diagnosis of COVID-19 (meaning PCR requirement, positivity or radiological/clinical findings) by percentage (%) in the patient profile applying to outpatient clinic that you encounter in your daily practice? | 0-20% | 27 (62.8%) | 10 (23.3%) | 6 (14%) | 0.189^b^ |
|  | 21-40% | 20 (57.1%) | 10 (28.6%) | 5 (14.3%) |  |
|  | 41-60% | 27 (62.8%) | 7 (16.3%) | 9 (20.9%) |  |
|  | 61-80% | 26 (52%) | 16 (32%) | 8 (16%) |  |
|  | 81-100% | 27 (71.1%) | 11 (28.9%) | 0 (0%) |  |
| Considering the normalisation period started after vaccination, evaluate the monthly non-COVID-19 case diversity between 1 and 10 by comparing it to the pre-pandemic period (before March 2020). 1:Only COVID-19, 10:Wide variety of cases | | 8 (1-10) | 7 (2-10) | 7 (4-10) | 0.210^a^ |
| Do you think that vaccination facilitates the patient diagnosis and follow-up processes during the COVID-19 pandemic? 1:not at all, 5:definitely yes | | 5 (1-5) | 4 (1-5) | 5 (1-5) | 0.179^a^ |
| With vaccination and normalization, has there been an increase in **appointments** for non-COVID-19 **diseases**? | Yes | 91 (61.5%) | 38 (25.7%) | 19 (12.8%) | 0.781^b^ |
|  | No | 20 (57.1%) | 11 (31.4%) | 4 (11.4%) |  |
| Do you think that there is an increase negligence of the follow-up of various diseases and/or complication rates due to COVID-19 pandemic? Score between 1 and 10. (1:Absolutely no 10:Absolutely yes) | | 7 (1-10) | 7 (1-10) | 6 (1-10) | 0.868^a^ |
| Do you think that there is an increase in the rate of laboratory and radiological tests required for diagnosis/follow-up of patients after normalization compared to pre-pandemic (March 2020)? (0:Certainly not, 10:Certainly yes) | | 7 (1-10) | 8 (1-10) | 8 (2-10) | 0.467^a^ |
| Do you think that after the normalization, the clinical services, the number of appointments and the hospital facilities are sufficient for the diagnosis and follow-up of the patients before the pandemic (March 2020)? (0:Certainly not, 10 Certainly yes) | | 6 (1-10) | 5.5 (1-10) | 6 (1-10) | 0.442^a^ |
| Evaluate the patient-physician communication by comparing it with the pre-pandemic (March 2020). | More positive communication than before the pandemic | 18 (66.7%) | 6 (22.2%) | 3 (11.1%) | 0.531^b^ |
|  | More negative/problematic communication than before the pandemic | 43 (53.8%) | 23 (28.7%) | 14 (17.5%) |  |
|  | Similar or same communication as before the pandemic | 66 (64.7%) | 25 (24.5%) | 11 (10.8%) |  |
| Evaluate the **compliance** of patients who require chronic and/or routine screening (cancer, diabetes, hypertension, asthma, COPD, etc.) from March 2020 to the present, to apply to the hospital or to comply with the controls, on a scale of 1 to 10, according to the majority. (1:No control visits 10: All controls have been done without interruption) (median,min-max)(mean$\pm SD$) | | 4 (1-9) | 4 (1-9) | 5 (1-9) | 0.098^a^ |
| Do you think that the hospital services and personal protective equipment provided by your hospital are sufficient? (1: Not at all, 5:Definitely yes) | | 4 (1-5) | 3.5 (1-5) | 3.5 (1-5) | 0.553^a^ |
| How do you think a COVID-19-centered clinical practice, which is implemented during the pandemic, affects field physicians mentally during their diagnostic processes? | The mental health of physicians were not affected. | 5 (62.5%) | 2 (25%) | 1 (12.5%) | 0.408^b^ |
|  | The mental health of physicians were affected positively. | 10 (83.3%) | 0 (0%) | 2 (16.7%) |  |
|  | The mental health of physicians were adversely affected. | 99 (57.9%) | 48 (28.1%) | 24 (14%) |  |

a:Kruskall-Wallis h test;b: Chi-Square Test;**:p<0.05. Numeric variables that are not normally distributed are median (min-max); categorical variables are presented as numbers (%lines).

**Table 3.** **Relationship between telemedicine services, chronic disease follow-up and potential causes of interruptions in the healthcare delivery**

| **Variables** | | **Does your institution/medical unit use telemedicine services in its routine clinical procedures?** | | | | |
| --- | --- | --- | --- | --- | --- | --- |
|  |  | **Very often** | **Sometimes** | **Never** | **p** |  |
| I think that the diagnosis and follow-up of chronic diseases are missed from time to time. | Yes | 27 (22%) | 36 (29.3%) | 60 (48.8%) | 0,505^a^ |  |
|  | No | 21 (24.4%) | 30 (34.9%) | 35 (40.7%) |  |  |
| I think that the diagnosis and follow-up of chronic diseases are significantly affected. | Yes | 42 (23.5%) | 57 (31.8%) | 80 (44.7%) | 0.852^a^ |  |
|  | No | 6 (20%) | 9 (30%) | 15 (50%) |  |  |
| Difficulty of finding an appointment | | 19 (25.7%) | 15 (20.3%) | 40 (54.1%) | **0.032^a^*** |  |
| Idea that health-care centers carry a higher risk of transmitting COVID-19 | | 36 (21.7%) | 55 (33.1%) | 75 (45.2%) | 0.547^a^ |  |
| **Inability** or hesitation to visit hospitals during the pandemic | | 26 (20.2%) | 40 (31%) | 63 (48.8%) | 0.360^a^ |  |
| Lack of control/follow-up | | 30 (20.7%) | 47 (32.4%) | 68 (46.9%) | 0.499^a^ |  |
| Increase in late diagnoses | | 28 (20.6%) | 38 (27.9%) | 70 (51.5%) | 0.058^a^ |  |

a: Chi-square test. Categorical variables are presented as numbers (%lines);*:p<0.05
